# Lack of racial and ethnic-based differences in acute care delivery in intracerebral hemorrhage

**DOI:** 10.1186/s12245-021-00329-w

**Published:** 2021-01-19

**Authors:** Chun Mei Su, Andrew Warren, Cassie Kraus, Wendy Macias-Konstantopoulos, Kori S. Zachrison, Anand Viswanathan, Christopher Anderson, M. Edip Gurol, Steven M. Greenberg, Joshua N. Goldstein

**Affiliations:** 1 Department of Emergency Medicine, Massachusetts General Hospital, Zero Emerson Place, Suite 3B, Boston, MA 02114 USA; 2 Department of Neurology, Massachusetts General Hospital, Zero Emerson Place, Suite 3B, Boston, MA 02114 USA

**Keywords:** Ethnic groups, Healthcare disparities, Intracranial hemorrhage, Emergency medical services, Acute care, Stroke

## Abstract

**Background and aim:**

Early diagnosis and treatment of intracerebral hemorrhage (ICH) is thought to be critical for improving outcomes. We examined whether racial or ethnic disparities exist in acute care processes in the first hours after ICH.

**Methods:**

We performed a retrospective review of a prospectively collected cohort of consecutive patients with spontaneous primary ICH presenting to a single urban tertiary care center. Acute care processes studied included time to computerized tomography (CT) scan, time from CT to inpatient bed request, and time from bed request to hospital admission. Clinical outcomes included mortality, Glasgow Outcome Scale, and modified Rankin Scale.

**Results:**

Four hundred fifty-nine patients presented with ICH between 2006 and 2018 and met inclusion criteria (55% male; 75% non-Hispanic White [NHW]; mean age of 73). In minutes, median time to CT was 43 (interquartile range [IQR] 28, 83), time to bed request was 62 (IQR 33, 114), and time to admission was 142 (IQR 95, 232). In a multivariable analysis controlling for demographic factors, clinical factors, and disease severity, race/ethnicity had no effect on acute care processes. English language, however, was independently associated with slower times to CT (*β* = 30.7 min, 95% CI 9.9 to 51.4, *p* = 0.004) and to bed request (*β* = 32.8 min, 95% CI 5.5 to 60.0, *p* = 0.02). Race/ethnicity and English language were not independently associated with worse outcome.

**Conclusions:**

We found no evidence of racial/ethnic disparities in acute care processes or outcomes in ICH. English as first language, however, was associated with slower care processes.

## Background

Between 40,000 and 67,000 people suffer from primary intracerebral hemorrhage (ICH) in the USA each year [1]. ICH accounts for about 10 to 15% of all stroke cases and is associated with higher mortality and disability than other stroke subtypes [2]. Roughly half of the mortality occurs within 24 h after onset [1], and so early treatment is thought to be critical [1].

In many hospitals, the initial management of ICH takes place in the prehospital and emergency department (ED) setting. Guidelines from the American Heart Association (AHA) recommend a time to computerized tomography (CT) scan for suspected stroke within 25 min of ED arrival and time to inpatient stroke unit admission within 3 h of arrival [3, 4]. Admission to a dedicated neuroscience intensive care unit (NeuroICU) seems to improve outcomes [5], suggesting that care by specialized providers adds substantial value.

Multiple studies have found that race and ethnicity are associated with worse care delivery and clinical outcomes. Minority patients have been found to receive slower or worse acute care in mild traumatic brain injury [6], acute asthma [7], and stroke [8, 9]. However, it is not clear whether those presenting with ICH, a stroke subtype with an often acute and clear change in neurologic function, are subject to the same potential biases in clinical management that may be found in other disease processes. Because of the importance of early and efficient treatment [1] and a paucity of prior research examining the acute care processes in ICH among different racial and ethnic groups, it is important to determine whether disparities exist in this area, and if so whether they are associated with worse outcomes.

## Methods

### Aims

This study aimed to determine whether proxies of acute time-sensitive care delivery in ICH differ by race and ethnicity. We selected specific metrics that mark critical times in care delivery, including time from ED arrival to initial diagnosis (CT scan), time from CT scan to inpatient bed request, and time from inpatient bed request to hospital admission. Additionally, between group differences in clinical outcomes such as Glasgow Outcome Scale (GOS), modified Rankin Scale (mRS), and mortality were examined.

### Study design

A retrospective review of a prospectively collected cohort of consecutive patients with ICH presenting to a single large urban tertiary care center was performed. Our hospital’s Institutional Review Board approved this study.

### Data collection

Patients were captured in an ongoing prospective cohort of consecutive patients presenting with primary ICH [10–12]. Patients are followed up for clinical outcomes, including phone calls, medical record review, and searches of the Social Security Death Index. Race and ethnicity were based on the patient’s self-report of their identity. Outcomes included discharge GOS score, mRS score, and 90-day mortality. An electronic database for the ED was queried for arrival day of week, hour of day, time of bed request and admission, and health insurance type.

### Study population

**Fig. 1:**
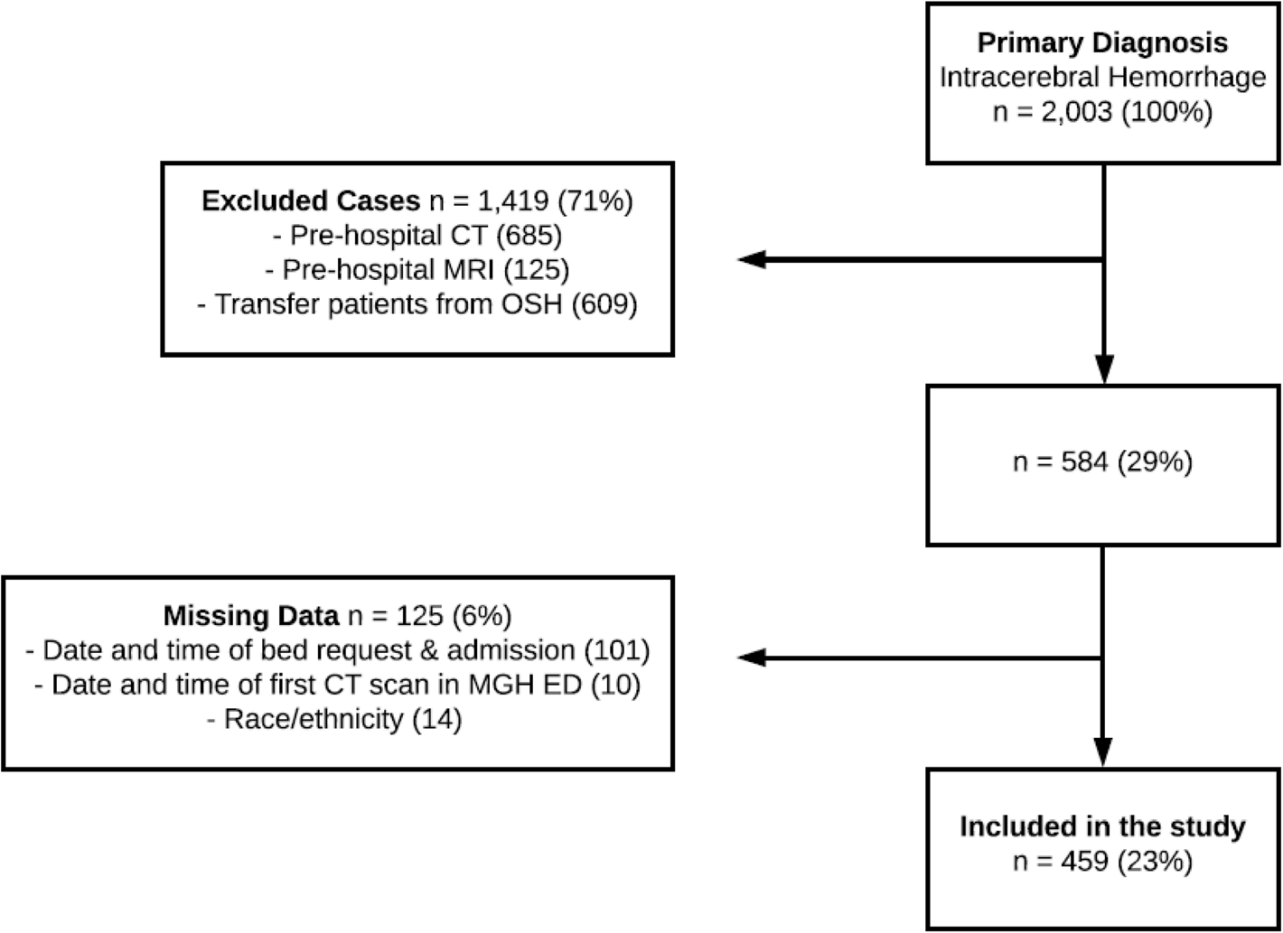
Flow diagram indicating selection of the study population. Note that these categories are not mutually exclusive. Percentages shown were rounded to the nearest percentage point

Patients aged 18 or greater presenting between January 1, 2006, and December 31, 2018, with primary spontaneous ICH were included. Exclusion criteria included secondary ICH (e.g., ICH associated with trauma, aneurysm, vascular malformation, tumor, etc.), patients transferred from an outside hospital with a diagnosis or those with prior diagnostic information, missing race, missing date/time of ED arrival, or missing date/time of CT scan (Fig. 1).

### Predictor variables

As there were small numbers of patients to analyze for some races and ethnicities, we dichotomized patients into two groups: non-Hispanic White (NHW) vs. minorities. The intent was to maximize our ability to detect any effect of minority status. We found the number of patients in each racial and ethnic category too small for robust analyses. When race was known but ethnicity unknown, we operationally imputed those for whom English was not a first language into the minorities category. There were 19 patients whose race was known but ethnicity unknown. Of these, two were coded as NHW and 17 as minorities based on whether English was their first language. Day of arrival was dichotomized into weekday or weekend, and time of arrival was categorized as day (7:00 AM–3:00 PM), evening (3:00 PM–11:00 PM), and overnight (11:00 PM–7:00 AM). In order to attempt to control for socioeconomic status, health insurance status was categorized as commercial, government, and other. In order to control for any effect of limitations in care, we captured any Comfort Measures Only (CMO) orders made within 24 h of ED arrival. These patients were not excluded because time to initial diagnosis was a primary analysis, and the CMO decision is often made only after the initial diagnosis and treatment. Its inclusion allowed us to explore any potential racial or ethnic differences in use of CMO orders.

### Statistical analysis

Descriptive statistics are presented as means and standard deviations for continuous variables and as frequencies and percentages for categorical variables. For the three time-to-event outcome variables, medians and interquartile ranges (IQRs) are presented due to their right-skewed distributions. The NHW group was compared to the minorities group using chi-square tests for categorical variables, two-tailed *t* tests for normally distributed continuous variables, and Wilcoxon rank-sum tests for nonparametric continuous variables. A power calculation was not performed a priori; given our final sample size, we had 80% power to detect a 14-min difference in time to CT scan between NHW and minorities at the *p* < 0.05 level.

Multiple linear regression analyses were conducted to determine the adjusted association between two racial/ethnic groups and the time-to-event outcome variables after controlling for potential confounders. The *β* coefficients were interpreted as the difference in minutes in the time-to-event outcome variables in response to every one-unit increase in the predictor variable, while holding all of the other confounders constant.

Due to the right-skewed distributions of all three time-to-event outcome variables, these outcome data underwent a natural log transformation, after which their distributions became fairly normal and unimodal. Multiple linear regression analyses were conducted on the three log-transformed outcome variables, and the results were consistent with those of the non-transformed outcome variables. For ease of interpretation of the results, the non-log-transformed outcome results are presented in the tables. Age is presented per decade (rather than per year) for ease of interpreting the odds ratio.

Observations with incomplete or missing information were removed from the analyses (see Fig. 1). All statistical analyses were performed using R statistical software version 3.6.3 (the R Foundation for Statistical Computing). A two-sided *p* value of < 0.05 was considered statistically significant.

## Results

### Participant and clinical characteristics

During the study period, 2003 patients presented with spontaneous primary ICH, of whom 459 met inclusion criteria (Fig. 1). The majority of patients excluded were due to presentation at another hospital first (and thus, initial diagnosis was already known upon arrival), and missing admission date/time for those presenting during a change in our electronic medical record system. Demographics are shown in Table 1.

**Table 1:** Demographics and clinical characteristics in the cohort

Variable	Overall	Non-Hispanic White	Minorities	***p*** value
**No. (%)**	459 (100%)	346 (75%)	113 (25%)	< 0.001**
**Age (years)**	73 (13.0)	75 (11.6)	68 (15.1)	< 0.001**
**Male (%)**	251 (55%)	188 (54%)	63 (56%)	0.88
**Race (%)**				< 0.001**
White	366 (80%)	346 (100%)	20 (18%)	–
Black	43 (9%)	0 (0%)	43 (38%)	–
Asian	38 (8%)	0 (0%)	38 (34%)	–
Hispanic	2 (0%)	0 (0%)	2 (2%)	–
Other	8 (2%)	0 (0%)	8 (7%)	–
Native Hawaiian/Pacific Islander	0 (0%)	0 (0%)	0 (0%)	–
American Indian/Alaskan Native	1 (0%)	0 (0%)	1 (1%)	–
More than one race	1 (0%)	0 (0%)	1 (1%)	–
Unknown	0 (0%)	0 (0%)	0 (0%)	–
**Ethnicity (%)**				< 0.001**
Hispanic	30 (7%)	0 (0%)	30 (25%)	–
Non-Hispanic	369 (80%)	296 (86%)	73 (65%)	–
Unknown	60 (13%)	50 (14%)	10 (9%)	–
**English is native language (%)**				< 0.001**
Yes	289 (63%)	257 (74%)	32 (28%)	–
No	87 (19%)	29 (8%)	58 (51%)	–
Unknown	83 (18%)	60 (17%)	23 (20%)	–
**Day of ED arrival (%)**				1.0
Weekday	326 (71%)	246 (71%)	80 (71%)	–
Weekend	133 (29%)	100 (29%)	33 (29%)	–
**ED arrival hour of day (%)**				0.02*
Day (7:00 am–3:00 pm)	206 (45%)	166 (48%)	40 (35%)	–
Evening (3:00 pm–11:00 pm)	181 (39%)	134 (39%)	47 (42%)	–
Overnight (11:00 pm–7:00 am)	72 (16%)	46 (13%)	26 (23%)	–
**Health insurance (%)**				0.22
Commercial	142 (31%)	105 (30%)	37 (33%)	–
Government	306 (67%)	235 (68%)	71 (63%)	–
Other	11 (2%)	6 (2%)	5 (4%)	–
**Initial GCS, median [IQR]**	13 [7]	14 [7]	13 [7.2]	0.62
**Discharge GOS, median [IQR]**	3 [2]	3 [2]	3 [2]	0.15
**Discharge mRS, median [IQR]**	5 [2]	5 [2]	4 [2]	0.05*
**In-hospital mortality (%)**	166 (36%)	136 (39%)	30 (27%)	0.02*
**90-day mortality (%)**	206 (45%)	165 (48%)	41 (36%)	0.04*
**Comfort Measures Only (CMO) status (%)**				0.19
Made CMO within 24 h of ED arrival	45 (10%)	38 (11%)	7 (6%)	–
Not made CMO within 24 h of ED arrival	414 (90%)	308 (89%)	106 (94%)	–
**ED arrival to CT scan (minutes), median [IQR]**	43 [55]	45 [59]	40 [47]	0.29
**CT scan to bed request (minutes), median [IQR]**	62 [81]	66.5 [89.5]	50 [56]	0.005*
**Bed request to admission (minutes), median [IQR]**	142 [137]	142.5 [147.2]	139 [113]	0.49

Data are represented as *n* (%), mean (SD), or median [IQR]. Percentages with decimal places might not add up to exactly 100% due to rounding. Because the distributions for ED arrival to CT scan time, CT scan to bed request time, and bed request to admission time are right-skewed, the median and IQR are presented here

*GCS* Glasgow Coma Scale, *GOS* Glasgow Outcome Scale, *mRS* modified Rankin Scale

*p < 0.05

**p < 0.01

There were no differences between the NHW and minorities groups in day of ED arrival (*p* = 1.0) or type of health insurance coverage (*p* = 0.22). However, the time of ED arrival differed among the two racial/ethnic groups, with the NHW group disproportionately presenting during the day and the minorities group disproportionately presenting during evening (*p* = 0.02). Both groups had similar disease severity on arrival as measured by initial Glasgow Coma Scale (GCS) score (NHW = 14 vs. minorities = 13, *p* = 0.62) and discharge outcomes as measured by GOS score (NHW = 3 vs. minorities = 3, *p* = 0.15). However, at discharge, the NHW group had greater disability as measured by discharge mRS (NHW = 5 vs. minorities = 4, *p* = 0.05) and higher 90-day mortality (48% vs. 36%, *p* = 0.04). Perhaps related to this, the NHW group was also more likely to be made CMO in the first 24 h after arriving in the ED compared to the minorities group (11% vs. 6%, *p* = 0.19) (Table 1).

### Association between race/ethnicity and processes of care

Compared with the minorities group, the NHW group had a similar median time from arrival to CT scan (45 vs. 40 min, *p* = 0.29) and time from bed request to admission (142.5 vs. 139 min, *p* = 0.49), but a longer time from CT scan to bed request (66.5 vs. 50 min, *p* = 0.005) (Table 1).

To control for potential confounders, multivariable analyses were performed. There were no statistically significant differences between NHW and minority patients in time to CT scan (*β* = − 10.8 min, 95% CI − 32.0 to 10.3, *p* = 0.31, Table 2), time to bed request (*β* = 1.1 min, 95% CI − 27.0 to 29.1, *p* = 0.94, Table 3), and time to admission (*β* = − 6.5 min, 95% CI − 62.4 to 49.5, *p* = 0.82, Table 4).

**Table 2:** Multiple linear regression model of time from ED arrival to CT scan

Variable	***β*** (95% CI)	***p***
**Non-Hispanic White**	− 10.8 (− 32.0, 10.3)	0.31
**Age per decade**	2.8 (− 3.7, 9.3)	0.40
**Male**	− 7.8 (− 22.7, 7.0)	0.30
**English as first language**	30.7 (9.9, 51.4)	0.004
**Initial GCS**	3.5 (1.6, 5.3)	0.0002
**ED arrival hour: day (7 am–3 pm)**	1.9 (− 8.3, 12.0)	0.71
**Weekday**	11.8 (− 4.5, 28.1)	0.15
**Commercial insurance**	10.7 (− 4.9, 26.3)	0.18
**Comfort Measures Only (within 24 h of ED arrival)**	− 20.3 (− 48.2, 7.7)	0.15
**Model** ***R***^**2**^	0.06655	
***F***	3.907

**Table 3:** Multiple linear regression model of time from CT to bed request

Variable	***β*** (95% CI), minutes	***p***
**Non-Hispanic White**	1.1 (− 27.0, 29.1)	0.94
**Age per decade**	6.1 (− 2.4, 14.6)	0.16
**Male**	− 7.0 (− 26.8, 12.7)	0.49
**English as first language**	32.8 (5.5, 60.0)	0.02
**Initial GCS**	4.0 (1.6, 6.4)	0.001
**ED arrival hour: day (7 am–3 pm)**	− 7.7 (− 21.1, 5.7)	0.26
**Weekday**	10.5 (− 10.8, 31.9)	0.33
**Commercial insurance**	− 1.1 (− 21.8, 19.6)	0.91
**Comfort Measures Only (within 24 h of ED arrival)**	2.8 (− 34.4, 40.0)	0.88
**Model** ***R***^**2**^	0.05151	
***F***	3.082

**Table 4:** Multiple linear regression model of time from bed request to admission

Variable	***β*** (95% CI), minutes	***p***
**Non-Hispanic White**	− 6.5 (− 62.4, 49.5)	0.82
**Age per decade**	2.0 (− 15.2, 19.2)	0.82
**Male**	− 32.9 (− 72.5, 6.7)	0.10
**English as first language**	42.2 (− 12.7, 97.2)	0.13
**Initial GCS**	3.0 (− 1.9, 7.8)	0.23
**ED arrival hour: day (7 am–3 pm)**	− 2.6 (− 29.6, 24.4)	0.85
**Weekday**	64.0 (20.7, 107.3)	0.004
**Commercial insurance**	1.3 (− 40.5, 43.1)	0.95
**Comfort Measures Only (within 24 h of ED arrival)**	25.6 (− 49.4, 100.7)	0.50
**Model** ***R***^**2**^	0.01943	
***F***	1.797	

However, English as first language and initial GCS independently predicted slower time to CT scan and bed request. Patients who were native English speakers waited longer for CT scan (*β* = 30.7 min, 95% CI 9.9 to 51.4, *p* = 0.004, Table 2) and bed request (*β* = 32.8 min, 95% CI 5.5 to 60.0, *p* = 0.02, Table 3). In addition, every unit increase in the initial GCS score was associated with a 3.5-min longer time to CT (*β* = 3.5 min, 95% CI 1.6 to 5.3, *p* = 0.0002, Table 2) and a 4.0-min longer time to bed request (*β* = 4.0-min, 95% CI 1.6 to 6.4, *p* = 0.001, Table 3). The only independent predictor of longer time from bed request to admission was arrival on weekdays vs. weekends (*β* = 64.0 min, 95% CI 20.7 to 107.3, *p* = 0.004, Table 4),

### Clinical outcome

To explore the associations between long-term clinical outcome, race/ethnicity, and acute processes of care, an exploratory multiple logistic regression analysis was conducted. In the multiple, adjusted model, neither race/ethnicity nor any of the acute processes of care significantly predicted the 90-day mortality rate (Table 5).

**Table 5:** Multiple logistic regression model of 90-day mortality

Variable	OR (95% CI), minutes	***p***
**Non-Hispanic White**	0.95 (0.85, 1.08)	0.44
**Age per decade**	1.11 (1.07, 1.15)	< 0.0001
**Male**	1.12 (1.03, 1.22)	0.01
**English as first language**	1.05 (0.93, 1.18)	0.44
**Initial GCS**	0.95 (0.94, 0.96)	< 0.0001
**ED arrival hour: day (7 am-3 pm)**	1.0 (0.94, 1.06)	0.98
**Weekday**	1.03 (0.94, 1.13)	0.54
**Commercial insurance**	0.99 (0.91, 1.09)	0.88
**Comfort Measures Only (within 24 h of ED arrival)**	1.49 (1.27, 1.75)	< 0.0001
**CT scan to bed request**	1.0 (1.0, 1.0)	0.84
**Bed request to admission**	1.0 (1.0, 1.0)	0.84

*OR* odds ratio, *CI* confidence interval, *GCS* Glasgow Coma Scale

## Discussion

Overall, we found no evidence for systematic differences in timing of acute ICH care processes for minorities. If anything, it appears that native English speakers may have slower times, and the reasons for this are unclear. It may be that in patients with acute ICH, our ED processes of care are streamlined enough to minimize any biases based on race or ethnicity.

For ICH, multiple studies have found greater disease incidence, severity, and mortality for minorities and Hispanics compared to non-Hispanic whites [13–15]. However, some have found better outcomes in minorities [16]. The causes are not clear, nor whether there are any disparities in acute stroke care and treatment. Findings vary across care settings, geographic areas, and treatment procedures [15, 17]. For instance, blacks and Hispanics with stroke symptoms have been found to suffer longer waiting times to see a physician in the ED [8, 9]. In contrast, Morris et al. found no evidence for racial disparities in time to CT scan in acute stroke, similar to our findings in ICH [18]. It may be that in ICH, a disease that typically leads to an acute and clear change in neurologic function, any effects of race and ethnicity on time to evaluation and neuroimaging are minimized.

One surprising finding was that non-Hispanic whites showed a longer wait for bed request. This effect disappeared when controlling for English as first language, suggesting that it reflects language rather than race or ethnicity. There are multiple possible explanations for this. First, it may be that non-native English speakers truly do receive more rapid care. Our healthcare providers may give extra attention to non-native English speakers resulting in faster movement. Alternatively, or in combination, English-speaking patients who are able to explain their symptoms and medical history effectively create an artificial barrier that slows down clinical processes. Language barriers may make healthcare providers spend less time interviewing the patient or family and more rapidly move to CT and admission. Second, it may be that there is no true effect, but unmeasured clinical confounders accounted for this finding. For example, if our analysis did not fully account for important differences in disease severity, minorities may have presented with more severe disease and received more rapid care because of this. Similarly, longer bed request times among English-speaking patients may reflect lengthier conversations with English-speaking patients and their families about the diagnosis, prognosis, and code status to determine intensive care unit (ICU) vs. floor bed request.

Interestingly, CMO status in the first 24 h of ED arrival was not associated with any acute processes of care. Past findings have shown that white patients with stroke are more likely to pursue CMO and withdraw from life-sustaining interventions compared to minorities [19–21] and that CMO status may lead to slower care. Contrary to the past findings, we did not find patients made CMO within 24 h of ED arrival to have received slower care. Our attempt to account for the confounding effects of CMO status was imperfect. We did not have available data on the time of CMO status to account for those who were made CMO prior to a particular acute care process; we only had data on the CMO status date.

The study has several limitations. First, our findings may not be generalizable or representative of the broader ICH population in the USA [14, 22]. Second, we did not perform individual analyses of all of the possible racial and ethnic subgroup combinations given the small number of patients in each subgroup. The small numbers in each group may have led to the loss of power to detect a significant effect in care on one or many minorities. Third, our analysis might have inadequately accounted for disease severity. It could be the case that race/ethnicity influences how quickly patients receive acute care in the ED only in patients with milder or ambiguous  neurological symptoms, where implicit bias is most likely to creep in [23], rather than in patients with severe disease presentation where it is clear that acute care must be deployed rapidly. In addition, some neurologic deficits can lead to more vs. less clear diagnosis (altered mental status or confusion, for example, vs. hemiparesis), and our analysis could not control for this. Fourth, our analysis might have inadequately accounted for differences in socioeconomic status (SES). We attempted to control for this by examining health insurance status, but had no ability to capture other markers of SES. Future studies may better capture other SES related factors such as income, occupation, living situation, and educational level. Fifth, the study might be underpowered to detect real race/ethnic disparities. Sixth, we were unable to capture English competency, only information on whether English was the primary language. Many minorities categorized as non-English speakers in this analysis might speak English well.

## Conclusions

In conclusion, we found no evidence for racial or ethnic disparities in the acute care processes of patients with acute ICH. Further investigations with higher numbers of minority patients are indicated to confirm these findings. Future work should also investigate disparities occurring at the hospital level in a larger, representative sample across hospitals.

## Data Availability

The datasets used and/or analyzed during the current study are available from the corresponding author on reasonable request. Please contact the corresponding author for data requests.

## Abbreviations

AHA: American Heart Association
CMO: Comfort Measures Only
CT: Computerized tomography
ED: Emergency department
GCS: Glasgow Coma Scale
GOS: Glasgow Outcome Scale
ICH: Intracerebral hemorrhage
IQR: Interquartile range
ICU: Intensive care unit
mRS: Modified Rankin Scale
NeuroICU: Neuroscience intensive care unit
NHW: Non-Hispanic White
SES: Socioeconomic status
